# Magnetic Driven Two-Finger Micro-Hand with Soft Magnetic End-Effector for Force-Controlled Stable Manipulation in Microscale

**DOI:** 10.3390/mi12040410

**Published:** 2021-04-07

**Authors:** Dan Liu, Xiaoming Liu, Pengyun Li, Xiaoqing Tang, Masaru Kojima, Qiang Huang, Tatsuo Arai

**Affiliations:** 1 Beijing Institute of Technology, School of Mechatronical Engineering, Beijing 100081, China; 3120185104@bit.edu.cn (D.L.); 3120170094@bit.edu.cn (P.L.); 3120170098@bit.edu.cn (X.T.); qhuang@bit.edu.cn (Q.H.); tarai118@jcom.zaq.ne.jp (T.A.); 2 Department of Materials Engineering Science, Osaka University, Osaka 560-8531, Japan; kojima@cheng.es.osaka-u.ac.jp; 3 Center for Neuroscience and Biomedical Engineering, The University of Electro-Communications, Tokyo 182-8585, Japan

**Keywords:** micromanipulation, micronewton, force-controlled, magnetically driven, stable grasping

## Abstract

In recent years, micromanipulators have provided the ability to interact with micro-objects in industrial and biomedical fields. However, traditional manipulators still encounter challenges in gaining the force feedback at the micro-scale. In this paper, we present a micronewton force-controlled two-finger microhand with a soft magnetic end-effector for stable grasping. In this system, a homemade electromagnet was used as the driving device to execute micro-objects manipulation. There were two soft end-effectors with diameters of 300 μm. One was a fixed end-effector that was only made of hydrogel, and the other one was a magnetic end-effector that contained a uniform mixture of polydimethylsiloxane (PDMS) and paramagnetic particles. The magnetic force on the soft magnetic end-effector was calibrated using an atomic force microscopy (AFM) probe. The performance tests demonstrated that the magnetically driven soft microhand had a grasping range of 0–260 μm, which allowed a clamping force with a resolution of 0.48 μN. The stable grasping capability of the magnetically driven soft microhand was validated by grasping different sized microbeads, transport under different velocities, and assembly of microbeads. The proposed system enables force-controlled manipulation, and we believe it has great potential in biological and industrial micromanipulation.

## 1. Introduction

Micromanipulation technology provides a valuable tool for the biological and industrial field, especially in the manipulation of micro-objects [1,2], cell stiffness measurement [3], and micro-objects assembly [4,5]. In recent years, micromanipulation has been widely used to perform the pick-hold-place operation for microspheres [6,7] and biological cells [8,9]. In micromanipulation, only a few effective force feedback grasping systems can be produced, due to the challenges in measuring force in the micro-nano field. As is well known, stable manipulation of micro-objects places strict requirements on force information. Therefore, an effective micromanipulation system must satisfy the above requirements at the micro-scale.

Micromanipulation can be divided into contact and noncontact, according to the form of manipulation. The former is based on the direct physical impact on the micro-objects [10,11,12]. The contact micromanipulations have the advantages of flexibility, significant drive force, and wide operating range; but there are also some obstacles, such as direct damage to biological targets and adhesive phenomena between a microgripper and a micro-object. Noncontact manipulation uses long-rang force or the local energy field, such as flowing liquid [13], optical tweezers [14], electric field [15], and magnetic field [16,17,18,19], to manipulate micro-objects. Noncontact manipulation can avoid adhesion force and the direct damage caused by direct contact manipulation [20,21]. However, deficiencies of noncontact manipulation include dependence on the microfluidic control and limitations in flexibility and driving force.

In the contact manipulation system, various actuation methods were researched. Most of the contact micromanipulation was actuated by a stepping motor [22,23], pneumatic cylinders [24], and piezoelectricity [25,26], for which the effects of inner vibration, velocity instability, and high drive voltage cannot be ignored. In recent years, the magnetic field force has attracted attention for its wireless drive and large driving force, which can overcome hysteresis, inner vibration, and high drive voltage. Nevertheless, the magnetic field force can only drive magnetic particles, but not non-magnetic targets, like biological cells. On the other hand, contact manipulation could cause direct damage to bio-objects in the grasping process, so it is necessary to control the manipulation force precisely. Unlike the control and detection of contact force in a macro environment [27,28,29], the micromanipulator system has difficulty in measuring the manipulation force in micronewton or nanonewton levels.

**Figure 1:**
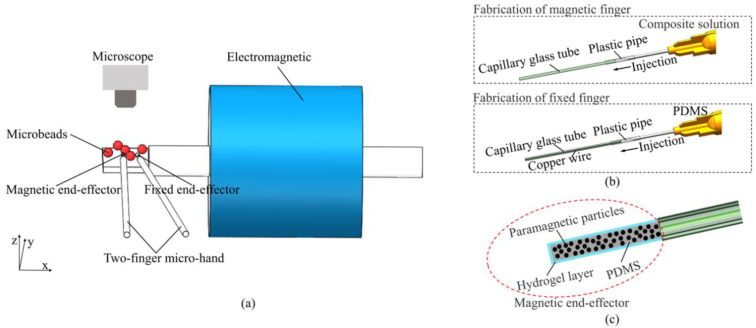
Schematic of the magnetically driven soft microhand. (**a**) Schematic of the manipulation system. (**b**) The fabrication method based on injection molding. (**c**) Illustration of the hydrogel skin formation onto the surface of the magnetic end-effector.

In this paper, we proposed a magnetically driven two-finger micro-hand with a soft end-effector, which provides force-controlled stable grasping manipulation. As shown in Figure 1a, the horizontal distance between the magnetic end-effector and the iron core of the electromagnet was 4.5 mm, and the movement of the magnetic end-effector was driven by magnetic field force. The magnetically driven micro-hand enables micronewton force-controlled clamping to achieve stable grasping. The performance of the magnetic end-effector was simulated, analyzed, and experimentally validated. The force-controlled manipulation was demonstrated by grasping different sized microbeads and transporting the microbeads under different velocities.

This paper is organized as follows: Section 2 introduces the configuration of the magnetically driven microhand. Section 3 analyzes the characteristics of the magnetic end-effector. The experiments and discussions are demonstrated in Section 4, and conclusions are presented in Section 5.

## 2. Overview of Magnetically Driven Two-Finger Microhand

### 2.1. System Setup

**Figure 2:**
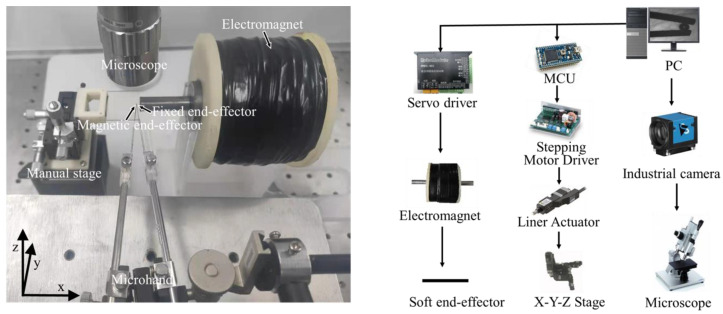
System setup.

As shown in Figure 2, the system we built in this paper mainly includes two parts: the motion control part and the vision part. The motion control part was comprised of a servo motor driver (RMDS-402, RoboModule, Shenzhen, China) that controls the input current on the electromagnet, a homemade electromagnet (the number of the turns is 4000) for generating the magnetic field, and a 12V switching power supply (LRS-350-24, MEAN WELL, Taiwan, China) to supply the power to the servo motor driver. A capillary glass tube with a diameter of 0.2 mm was utilized as a mold to produce a soft end-effector. The micro-hand was held by an X-Y-Z motorized stage (TAM-655, Sigmakoki, Japan) and three stepping motors (PKP523N12B, Orientalmotor, Japan) powered by stepping motor drivers (SG-55MA, Sigmakoki, Japan).

The vision part consists of monocular microscopy (CX-10C, Hirox, Japan) with a 140× objective lens, a digital camera (DFK 23U274, Imagingsource, Germany), and a high-speed camera (Fastcam MC-2, Photron, Japan) with a maximum frame rate of 2000 fps.

### 2.2. Magnetic Drive

The magnetic end-effector was driven by the magnetic field force generated by a customized electromagnet related to the current and the number of the copper wire turns. According to the electromagnetism formulas [30], the magnetic flux density, *B*, generated by an electromagnet in the applied current, *I*, is:

*B* = *μNI* (1)

where *μ* = *μ_τ_μ*_0_, *μ_τ_* is the magnetic permeability of iron core, *μ*_0_ is permeability of vacuum, *N* is the number of turns of the copper wire, and *I* is the current input to the electromagnet.

The magnetic end-effector was actuated by the magnetic field force. When applying a magnetic field in the workspace, the magnetic force *F_m_* is given by [19]:

*F_m_* = (*m* ∇) *B* (2)

where *m* is the magnetic moment of the magnetic end-effector.

### 2.3. Fabrication

Here, the fabrication technique was provided. The soft magnetic microhand consists of two end-effectors: a fixed end-effector and a magnetic end-effector that can be actuated by magnetic field excitation. The magnetic end-effector was created by embedding paramagnetic particles with a 5 μm diameter into polydimethylsiloxane (PDMS) solution. The mass ratio for the paramagnetic particles and PDMS was 1:1. The fixed end-effector was fabricated by PDMS and copper wire with 100 μm diameter. The copper wire was used to increase the elastic coefficient of the fixed end-effector. The copper wire could be changed to other materials with different diameters to achieve bigger or small working spaces.

First, the mixture solution (PDMS and paramagnetic particles) and the PDMS solution were squeezed into an empty capillary glass tube and a capillary glass tube with copper wire molds, respectively, through a syringe, as shown in Figure 1b. The molds were then placed in a 70 °C incubator box for 30 min. After curing the PDMS, the mold tips were sealed in hydrofluoric acid to melt the capillary glass, and the length of the end-effector was approximately 5 mm. Then, the soft end-effector was immersed in a hydrogel solution and exposed to ultraviolet (UV) radiation. As shown in Figure 1c, a thin film of hydrogel was generated on the surface of the soft end-effector, which can overcome adhesion force. Finally, the soft end-effector was immersed in octadecyldimethyl (3-trimethoxysilylpropyl) ammonium chloride solution (60% in methanol; ABCR GmbH & Co. KG, Shanghai, China) for silanization.

## 3. Testing and Analysis of Magnetic End-Effector Movement

### 3.1. End-Effector Movement Simulation

To analyze the movement of the end-effector generated by the magnetic field, the solid mechanics and magnetic fields were implemented to analyze the magnetic field strength and motion of the magnetic end-effector in finite element simulations. According to the magnetic properties and the structure of the electromagnet, a simple 3D model was constructed based on the actual parameters. The copper coil turns were set as 4000, and the driving current was 8 A.

**Figure 3:**
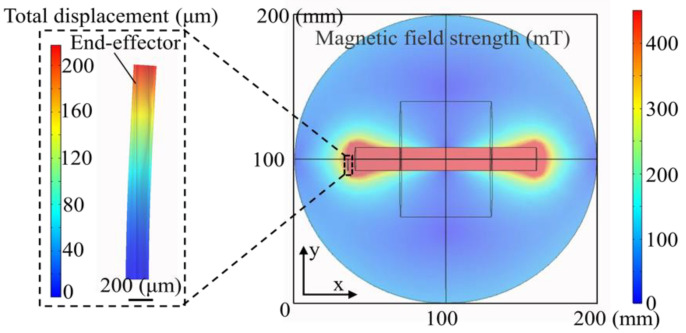
Finite element simulation of magnetic field distribution and tip displacement.

As shown in the simulation results of the magnetic field in Figure 3, the magnetic field strength decreased from the iron core area to the edge. The actual magnetic field strength generated by the electromagnet were measured with a Tesla meter, and the simulation results were consistent with the measured results. The displacement of the magnetic end-effector was shown in the partial magnification in Figure 3. The displacement simulation clearly showed the displacement of the magnetic end-effector was about 200 μm. It convinced us that micro-objects can be grasped with the proposed system.

### 3.2. Magnetic Force Calibration

#### 3.2.1. The Stiffness of the Magnetic End-Effector 

**Figure 4:**
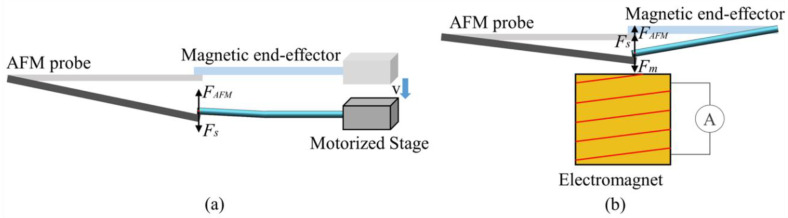
Schematic of the magnetic driven soft end-effector calculation. (**a**) Stiffness calibration of the magnetic end-effector. (**b**) Magnetic force measurement of the magnetic end-effector under an applied magnetic field.

The stiffness (*k_s_*) of the magnetic end-effector was measured using a reference AFM probe with an equivalent stiffness of *k_A_* = 2.8N/m (NSC18/AL BS). During the measurement, the reference probe was mounted on the sample platform horizontally, and the magnetic end-effector was utilized to compress the reference probe end by moving the X-Y-Z motorized stage a certain distance, Δz_s._ As shown in Figure 4a, the equilibrium equation can be obtained by:

(3) $$F_{AFM1}=F_{S1}$$

where *F_AFM_* is the interaction force between the magnetic end-effector and probe, and *F_s_* is the deformation force of the magnetic end-effector. According to Hooke’s law, *F_AFM_*_1_ and *F_s_*_1_ can be given by:

(4) $$F_{AFM1}=k_{A}\Delta z_{r1}$$

(5) $$F_{s1}=k_{e}\left(\Delta z_{s}-\Delta z_{r1}\right)$$

where Δ*z_r_*_1_ is the deflection of the reference probe that can be measured. From Equations (3)–(5), the stiffness of the magnetic end-effector, *k_s_*, can be calculated by:

(6) $$k_{s}=\frac{k_{A}\Delta z_{r1}}{\Delta z_{s}-\Delta z_{r1}}$$

Therefore, the stiffness of the magnetic end-effector was calculated to be 0.29 N/m

#### 3.2.2. Magnetic Force Test

The magnetic force (*F_m_*) of the magnetic end-effector was measured by using an AFM probe and an electromagnet. As shown in Figure 4b, the AFM probe and magnetic end-effector were mounted on the sample platform horizontally, and the vertical distance between the iron core of the electromagnet and the magnetic end-effector was 4.5 mm. Initially, we adjusted the location of the probe to make its surface nearly in contact with the magnetic end-effector. The magnetic field generated by applying a current, *I*, to the electromagnet forced the magnetic end-effector motion to compress the probe. By measuring the deflection (Δ*z_r_*_2_) of the probe, *F_m_* can be calculated by

(7) $$F_{m}=F_{AFM2}+F_{s2}$$

Furthermore, Equation (7) can be simplified as

(8) $$F_{m}=k_{A}\Delta z_{r2}+k_{s}\Delta z_{r2}$$

Therefore, the magnetic force on the magnetic end-effector produced by each current can be calculated according to Equation (8).

### 3.3. Grasping Analysis

**Figure 5:**
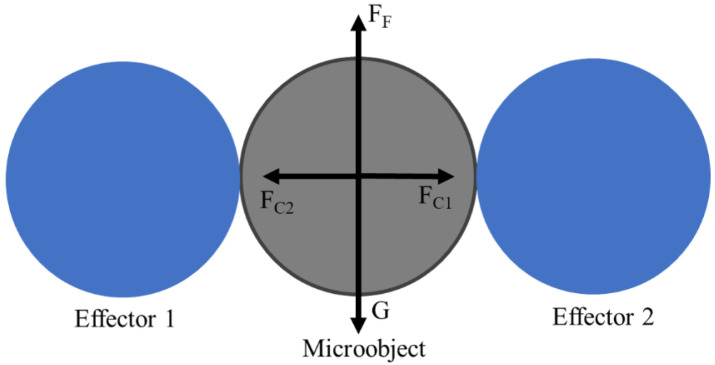
Forces acting on a microobject.

In this section, we analyzed the effect of the clamping force on grasping. In the analysis of grasping, we ignore the grasping angles because of the small inclination angle between the effector and the console. During grasping, the terms *G* (gravity), *F_F_* (friction), and *F_C_* (clamping force) are the main forces acting on the microobject, as shown in Figure 5. To grasp a microobject, a minimum amount of friction, *F_F_*, is required.

(9) $$F_{F}\geq G$$

*F_F_*, depending on *F_C_* acting on the microobject, should be made larger than *G*.

### 3.4. Characteristic Analysis

#### 3.4.1. Static Performance Analysis 

**Figure 6:**
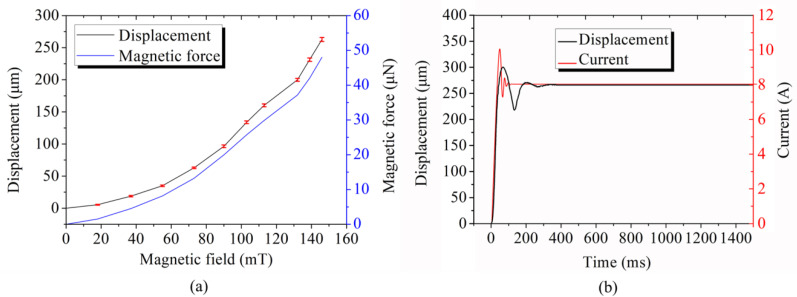
Motion analysis of the magnetic end-effector under a magnetic field. (**a**) The relationship between the magnetic field, displacement, and magnetic force; (**b**) The step response to the current of 8A.

In order to verify the theoretical and finite element models, displacement measurement experiments were conducted at different magnetic fields. Figure 6a shows the magnetic field-displacement-magnetic force test results. Ten tests were carried out at different magnetic fields, and the average value and the standard deviation of the displacement were measured. The displacement curve showed that the relationship between the magnetic field and displacement was nonlinear. The closer the magnetic end-effector was to the iron core, the greater the magnetic field gradient would be. The actual displacement was consistent with the simulated displacement. Besides, the standard deviations ranged from 0.61–3.13 μm. Furthermore, the deflection of the magnetic end-effector was 0.15 μm when the minimum current input to the electromagnet was 10 mA. Therefore, the resolution of the clamping force sensing was evaluated to be 0.48 μN from Equation (8).

The clamping force of each magnetic field was calculated according to Equation (8). Furthermore, the magnetic force curve was consistent with the displacement curve, and the magnitude of the magnetic force was between 0.48–48.01 μN. The micrometer operation accuracy and micronewton magnetic forces were sufficient for stable grasping and force-controlled manipulations.

#### 3.4.2. Dynamic Performance Analysis

In the control process, the step response reflects the stability of the manipulation system. An 8 A current was applied to the electromagnet, and the variation of the current and the displacement is shown in Figure 6b. The movement of the magnetic end-effector was registered by a high-speed camera with a frame rate of 2000 fps, and the current variation was given by the servo control system. As shown by the curves in Figure 6b, overshoots of the displacement together with the current have been found. The displacement of the magnetically driven end-effector reacheed a steady state after 350 ms.

## 4. Experiments and Discussion

### 4.1. Force-Controlled Grasping Manipulation

The experiments aimed to stablize grasping and transport of microbeads to the anticipant position. The X-Y-Z motorized stage was responsible for positioning and transportation, and the magnetically driven microhand was used for grasping and releasing operations. The transportation scope was determined by the liner actuator, which had a range of 15 mm. After focusing on the micro-objects and position of the microhand, the system began manipulation. Firstly, the micro-objects were placed between the two end-effectors. Secondly, the magnetic end-effector was driven to approach and grasp micro-objects by the magnetic force generated by applying a current to the electromagnet. Then, micro-objects were transported to the anticipated position by X-Y-Z motorized stage. Finally, the micro-objects were successfully released by the magnetically driven microhand.

**Figure 7:**
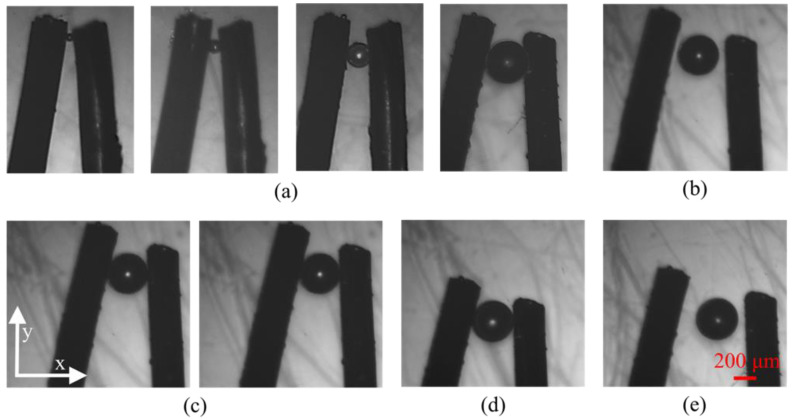
Micromanipulation of microbeads: (**a**) grasping microbeads with diameters of 50, 100, 200, and 300 μm; (**b**) Alignment; (**c**) Clamping microbeads; (**d**) Translating microbeads; (**e**) Releasing microbeads.

In this part, the force-controlled manipulation capability was demonstrated by operating the microbeads with diameters of 50 μm, 100 μm, 200 μm, and 300 μm, as shown in Figure 7a. A microbead with 300 μm diameter was taken as an example to perform the grasping, releasing, and transportation operation. The distance between the two tips was 400 μm. The experiments were carried out in a 1% sodium dodecyl sulfate (SDS) environment. The operation process can be described as follows.

1. Locate the microbead at the middle of two end-effectors by moving the X-Y-Z motorized stage driven by the stepping motor, as shown in Figure 7b.
2. The magnetic end-effector was moved in the direction of the microbead until both the two end-effectors nearly contacted the microbead by increasing current, *I*_1_. The magnetic end-effector was subjected to a magnetic force *F*(*I*_1_) at the current position.
3. Figure 7c shows the grasping process by increasing another current, *I*_2_, to grasp the microbead. The magnetic force on the magnetic end-effector was *F*(*I*_2_) at this moment. Therefore, the clamping force on the microbead was *F*(*I*_2_) − *F*(*I*_1_).
4. Transport the microbead to the desired location by moving the X-Y-Z motorized stage after grasping the microbead, as shown in Figure 7d.
5. Finally, move the magnetic end-effector to the initial location to release the microbead, as shown in Figure 7e.

Experiments on force-controlled grasping were demonstrated above. After the microbead was clamped, tit needed to be transported to the desired location. To discuss the effect of transporting velocity and clamping force on the transportation of the microbeads, experiments were conducted. During the experiments, the transportation of the microbead was controlled by X-Y-Z motorized stage.

**Figure 8:**
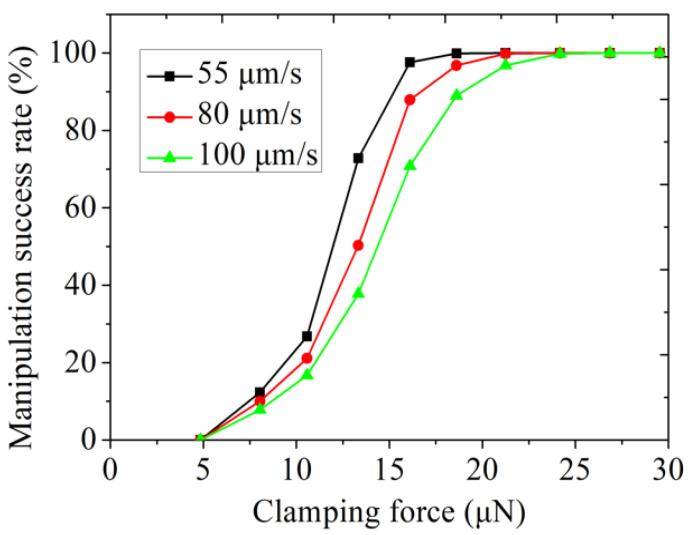
The relationship between clamping force and success rate of grasping microbeads.

We plotted the clamping force and manipulation success rate graph, where a sequence of transportation velocities (55 μm/s, 80 μm/s, and 100 μm/s) were used to transport a 300 μm microbead. As shown in Figure 8, we concluded that the microbead could be gripped when the clamping force exceeded 4.8 μN, because the friction caused by the clamping force of 4.8 μm was in equilibrium with gravity. Moreover, the transportation success rate increased monotonically when the clamping force increased, and a 100% success rate was achieved eventually. Notably, the three curves had a similar variation trend. During transportation, inner vibrations of the stage and motors can affect the transportation success rate. Experiment results demonstrated that the inner vibration can be completely overcome when the clamping force exceeded 24 μm. In this paper, a large clamping force will only increase the deformation of the soft end-effector, and it will not affect the transportation success rate. Therefore, the faster the velocity, the greater the clamping force that was required to achieve a 100% transportation success rate. A video of transporting 300 μm microbeads at different velocities is shown in the Supplementary Materials.

### 4.2. Assembly of Microbeads

**Figure 9:**
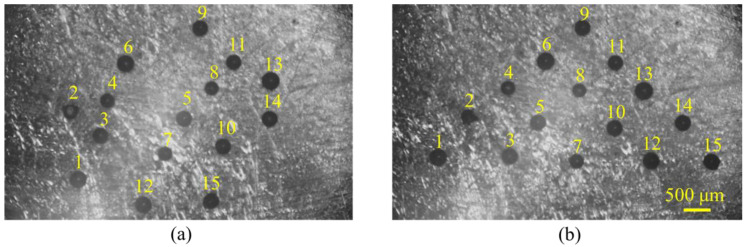
Assembly of the 2D pattern. (**a**) Optical microscopy image of collected 15 microbeads. (**b**) The assembled triangle pattern.

Before assembling, ten tests for grasp-transport-release of 300 μm microbeads were performed to analyze the position accuracy by measuring the position before and after release. The average error and position deviation of microbeads were 36 μm and 27 μm, respectively. To validate the microassembly manipulation capability of the proposed soft magnetic two-finger micro-hand, the microbeads with a 300 μm diameter were manipulated to form a two-dimensional arrangement, as shown in Figure 9. The entire assembly process was finished in about 5 min, and the average manipulation time of one microbead was about 20 s.

As shown in Figure 9, 15 microbeads were assembled in the triangular array. To avoid collision between the neighbor microbeads, the order of placing the microbeads was shown in the label because the microhand was on the left side of the workspace.

### 4.3. Discussion

The experimental results demonstrated that the proposed system, using the movement of the magnetic end-effector generated by the magnetic field, could achieve stable grasping and micronewton force-controlled manipulation. Using only one homemade electromagnet and a simple system setup meant the proposed method was simple and low cost. Grasping different sized microbeads indicated our system can operate various tasks. Transporting velocity and clamping force influencing the transportation of the microbeads were investigated by the experiments. Current is the only factor that controls the clamping force, and different clamping forces can be reached through changing the current. For non-biological targets, a large clamping force allowed for more stable manipulation.

Compared with conventional contact micromanipulators [10,11,12], the proposed system avoids direct damage to biological targets and adhesive phenomena and provides excellent dynamic characteristics and force-controlled manipulation. On the other hand, the magnetically driven system overcomes inner vibration generated by the stepping motor [22,23] and avoids the high drive voltage required by the piezoelectric [25,26]. Differing from the noncontact manipulation using fluid field force [13], optical tweezers [14], and electric field [15], the proposed system, based on the magnetic field force, provides flexibility and force-controlled manipulation of micro-objects, which causes little damage to biological micro-targets. Experiments of grasping, transporting, and assembling microbeads demonstrated our system could achieve a stable and force-controlled manipulation in the industrial and biological field.

In this research, the force-controlled manipulation feasibility of the soft magnetic end-effector was proved. However, there is still a restriction in micromanipulation. The soft two-finger microhand cannot operate small sized targets (less than 10 μm) flexibly because the size of the soft end-effector is large.

## 5. Conclusions

This paper proposed a two-finger micro-hand with a soft magnetic end-effector that can perform micronewton force-controlled micromanipulations. The magnetic field drives the magnetic end-effector and resulted in the excellent dynamic characteristics of the proposed system. The movement of the magnetic end-effector can be controlled by a current of 10 mA, and the clamping force can be as small as 0.48 μN. The experiments grasping microbeads of different sizes and transporting microbeads with different velocities demonstrated that the proposed microhand not only operated different tasks, but also performed force-controlled and stable grasping manipulations. The assembly of the triangle pattern indicated the proposed magnetically driven soft microhand has potential in biological and industrial applications.

In future, we will add visual feedback in the system to achieve automatic manipulation, and the proposed soft microhand will be applied to operate biological cells.

## Data Availability

Not applicable.

## Supplementary Materials

The following are available online at https://www.mdpi.com/article/10.3390/mi12040410/s1, Video S1: Transporting 300 μm microbeads at different velocities.
